# Hybrid Silica-Based Fillers in Nanocomposites: Influence of Isotropic/Isotropic and Isotropic/Anisotropic Fillers on Mechanical Properties of Styrene-Butadiene (SBR)-Based Rubber

**DOI:** 10.3390/polym13152413

**Published:** 2021-07-22

**Authors:** Mariapaola Staropoli, Vincent Rogé, Enzo Moretto, Joffrey Didierjean, Marc Michel, Benoit Duez, Pascal Steiner, Georges Thielen, Damien Lenoble, Jean-Sébastien Thomann

**Affiliations:** 1 MRT Department, Luxembourg Institute of Science and Technology, 41 Rue du Brill, Belvaux, L-4422 Luxembourg, Luxembourg; vincent.roge@list.lu (V.R.); enzo.moretto@list.lu (E.M.); joffrey.didierjean@list.lu (J.D.); marc.michel@list.lu (M.M.); damien.lenoble@list.lu (D.L.); jean-sebastien.thomann@list.lu (J.-S.T.); 2 Goodyear S.A, Avenue Gordon Smith, Colmar-Berg, L-7750 Luxembourg, Luxembourg; benoit_duez@goodyear.com (B.D.); pascal_steiner@goodyear.com (P.S.); georges_thielen@goodyear.com (G.T.)

**Keywords:** nanocomposites, silica, Sepiolite, dual fillers

## Abstract

The improvement of mechanical properties of polymer-based nanocomposites is usually obtained through a strong polymer–silica interaction. Most often, precipitated silica nanoparticles are used as filler. In this work, we study the synergetic effect occurring between dual silica-based fillers in a styrene-butadiene rubber (SBR)/polybutadiene (PBD) rubber matrix. Precipitated Highly Dispersed Silica (HDS) nanoparticles (10 nm) have been associated with spherical Stöber silica nanoparticles (250 nm) and anisotropic nano-Sepiolite. By imaging filler at nano scale through Scanning Transmission Electron Microscopy, we have shown that anisotropic fillers align only in presence of a critical amount of HDS. The dynamic mechanical analysis of rubber compounds confirms that this alignment leads to a stiffer nanocomposite when compared to Sepiolite alone. On the contrary, spherical 250 nm nanoparticles inhibit percolation network and reduce the nanocomposite stiffness.

## 1. Introduction

The improvement of mechanical properties of rubber-based nanocomposites has been extensively investigated over the last decades. The development of composites for aeronautic, space, marine, military, or automotive applications is one of the main efforts of many industries at the present time [1]. Particularly, industrials are interested in improving the stiffness, the modulus, the fatigue resistance, the wear resistance, the corrosion resistance, or the rolling resistance with cheap, lightweight, and non-toxic materials. As an example, the reinforcement of soft synthetic rubbers like styrene-butadiene rubber (SBR) is crucial for tire technology industries [2]. Polymer-based composites are usually reinforced with fillers and coupling agents [3]. Consequently, many different types of fillers have been investigated for the improvement of mechanical properties of SBR-based polymers. They can be classified in three categories: reinforcing fillers, semi-reinforcing fillers, and non-reinforcing fillers, according to their size, shape, and composition [4]. Reinforcing fillers are usually nanometric objects with one dimension smaller than 100 nm. They have a strong interaction with the polymeric matrix and result in an increase of the tensile strength, tear resistance, and abrasion resistance [5]. Semi-reinforcing fillers (100–500 nm) induce a moderate improvement of the tensile strength and tear resistance, but have no effect on the abrasion resistance [6]. Non-reinforcing fillers (>500 nm) have no specific effect on mechanical properties; they are most often used as diluent.

Several types of reinforcing fillers have been already investigated. Inorganic fillers represent a large fraction of them. We can list natural mineral clays, like montmorillonite [7], mica [8], talc [9]; salts, like calcium carbonate CaCO_3_ [10], Ca_2_SO_4_ [11], BaSO_4_ [12], phosphates [13]; and metal oxides or hydroxides, like SiO_2_ [14], ZnO [15], Al_2_O_3_ [16], MgO [17], Al(OH)_3_ [18], or Mg(OH)_2_ [19]. Organic fillers have also been studied as reinforcing fillers, like carbon-based materials: black carbon [20], graphite [21], and carbon nanotubes [22]; or bio-sourced cotton [23], wood flour [24], and cellulose fibres [25]. Among all cited reinforcing fillers, black carbon and silica nanoparticles are the most used for SBR rubber reinforcement in industrial applications [2,26,27]. Carbon black has been the first reinforcing filler largely used in the rubber industry owning to its ability to improve tear strength, hardness, and abrasion resistance of many rubbers [28]. Later, the development of “green tires” based on silica nanoparticles fillers has demonstrated enhanced reinforcing properties of silica on abrasion resistance, rolling resistance, and wet grip compared to black carbon [29]. The origin of the reinforcement in the composite arises from rigid SiO_2_ particles that act as stress concentrators due to their different elastic properties compared to the polymer matrix [30].

Usually, fumed or precipitated silica nanoparticles are used as silica fillers. If they are relatively cheap to process, those silica nanoparticles tend to agglomerate in the rubber, due to silanol surface group interactions forming inter-particles bonding [31] and show poor filler–rubber interactions [32]. Unfortunately, those two parameters are crucial to reach efficient homogeneous reinforcing properties. Consequently, different strategies have been foreseen in order to improve the processability of silica, like the use of processing oils [33] or silane coupling agents, to enhance the dispersity and chemical interaction between the rubber and fillers. Most of the coupling agents used in silica tread compounds belong to the families of organo-silane. The hydrophilic part of the silane coupling agent reacts with silanol surface groups of the silica [34], while the hydrophobic alkyl-mercapto (CH_2_)_n_-SH) will enhance silica dispersion and covalently react with the accelerator, sulphur, and finally rubbers during the vulcanization step [35]. Sol-gel synthesised silica nanoparticles represent another strategy to reach efficient silica-based reinforced particles. The most known process is based on the poly-condensation of a silicon alkoxide precursor, usually the tetraethyl orthosilicate (TEOS), in basic conditions. It is referred to as the Stöber process [36]. Some studies have shown that Stöber silica nanoparticles are more homogeneously dispersed in rubbers [37,38,39]. The benefit of the Stöber process is that it allows the control of particles sizes, which is another parameter affecting composites’ mechanical properties [14,30]. Other works have focused on the synthesis of silica nanoparticles in-situ during the composite processing. They highlight a homogeneous silica dispersion in the nanocomposite, even without silane coupling agent, with an improvement of the nanocomposite tensile strength [40,41,42].

In parallel to concentrated research efforts on spherical nanoparticle fillers, the interest in fillers’ morphological aspect has recently been growing in the scientific community. Anisotropic structures have emerged as a new possibility to further strengthen mechanical properties of rubber-based nanocomposites. R. Scotti et al. have synthesized anisotropic silica nanorods (with an aspect ratio up to 10) using a Stöber-derived process with CTAB as surfactant [43]. They pointed out a stronger reinforcement of the nanocomposite with anisotropic silica. This phenomenon is partially explained by an alignment of anisotropic filler domains oriented in the main axis where a high rubber fraction is tightly trapped between fillers [44]. This alignment is favoured with higher nanorod aspect ratios. The same behaviour has been observed with anisotropic silica-based natural clays: Sepiolite. Different studies have shown that Sepiolite could be an effective substitute to spherical silica nanoparticles [45]. As observed in the case of silica nanorods, the formation of oriented Sepiolite aggregates in the rubber matrix reinforce mechanical properties of composites [46]. However, a surface state modification (HCl treatment) of Sepiolites nanofibers is often performed in order to create more silanol surface reactive groups and to favour the filler/rubber interaction [47]. Another alternative to conventional fillers could be the use of hybrid fillers with identical or different morphologies [48]. Dual fillers nanocomposites based on black carbon and precipitated silica have been widely studied and used over the last decades, particularly in the tire industry [49,50]. Other studies have shown an interest in the synergetic effect of fillers with different morphologies, like spherical nanoparticles associated with 1D materials such as carbon nanotube [22], montmorillonite [51] or Sepiolite [48].

According to those previous works, it appears that the study of the reinforcement of a type of rubber filled with precipitated silica partially substituted by spherical and anisotropic silica structures could strengthen the understanding of the reinforcement mechanism. The novelty of this study is to elucidate the origin of this reinforcement, by substituting the fractal spherical silica filler with anisotropic Sepiolite, or bigger spherical nanoparticles. For the first time, this study highlights the direct correlation between the specific orientation of the fillers at the microscopic level and the resulting macroscopic mechanical properties measured through DMA and tensile tests. Consequently, in this work, we aim at studying the synergetic effect occurring between two types of hybrid silica-based fillers, fractal/spherical and fractal/anisotropic, on the mechanical properties of the synthesized nanocomposite. HDS particles, characterized by a fractal surface, have been used as main filler type and mixed with Sepiolite and Stöber particles, rod-like and spherical, respectively, for obtaining dual filler compounds. Highly Dispersed Silica nanoparticles (HDS)/Stöber silica nanoparticles and precipitated silica HDS/Sepiolite have been processed in a SBR-based rubber matrix.

## 2. Materials and Methods

All chemical used for the synthesis of sol-gel silica nanoparticles have been purchased from Sigma Aldrich. The growth of silica nanoparticles was performed as follows: in 1 l of ethanol (99%), 80 mL of ultra-pure water (>18 Ω·cm), and 45 mL of ammonium hydroxide solution (NH_4_OH 25%) were added at room temperature under stirring at 500 rpm. After homogenisation, 46 mL of Tetraethyl orthosilicate (TEOS 99%) were quickly added to the solution and left under stirring for 2 h. The grown silica nanoparticles were extracted from the solution by centrifugation at 2500 rpm for 10 min and cleaned in water and ethanol twice. This process allowed the synthesis of homogeneous and dispersed silica nanoparticles with a diameter of 250 nm.

High-resolution Scanning Transmission Electron Microscopy (STEM) images of nanocomposites were recorded with a Helios Nanolab 650 microscope (FEI, Eindhoven, Netherlands), at an acceleration voltage of 30 kV and a current of 50. STEM analyses were performed in bright field mode. Nanocomposites lamellas around 50–70 nm of thickness were prepared using a Cyro-Ultramicrotom EM UC7 (Leica, Wetzlar, Germany), at a temperature of −120 °C. Dynamic modulus of nanocomposites were determined using a 242C dynamic mechanical analyser (DMA) (Netzch, Selb, Germany). Thermograms were obtained in single cantilever mode, with a free length of 5 mm (15% strain amplitude) under a vibration frequency of 10 Hz. The studied temperature range was fixed between –100 °C to 8 °C with a heating rate of 0.5 °C∙min^−1^ in air atmosphere. The tensile behaviour of nanocomposites was determined at room temperature, under a strain rate of 3 mm·s^−1^, with a 5967 Series Universal Testing System (Instron, Norwood, MA, USA).

### Nanocomposite Synthesis Procedure

Nanocomposites based on SBR and PBD (polybutadiene) were compounded in a Brabender Plasti-Corder Lab station internal mixer, working with 85 cm^3^ mixing chamber and a 0.75 fill factor. The Goodyear Tire & Rubber Company (Colmar-Berg, Luxembourg) provided all raw materials: HDS nanoparticles (10 nm, 200 m^2^·g^−1^ silica grade), SBR rubber, polybutadiene rubber, zinc oxide, Treated Distillate Aromatic Extracted oil (TDAE oil), stearic acid, N-(1,3-Dimethylbutyl)-N′-phenyl-p-phenylenediamine (6PPD), Bis(triethoxysilylpropyl) disulphide (TESPD), sulfur, 2-Mercaptobenzothiazole (MBT), Diphenylguanidine (DPG), and N-Cyclohexyl-2-benzothiazolesulfenamide (CBS). The composition of composites (expressed in phr) is presented in Table 1. Typically, 80 phr of SBR and 20 phr of PBD were blended in the mixing chamber at 80 °C, 40 rpm. Then, 25 phr of TDAE oil, 2.5 phr of zinc oxide, 3 phr of stearic acid, 2.5 phr of 6PPD, 8 phr of TESPD, and 80 phr of silica fillers were introduced under mixing for 10 min. After this first step, the nanocomposites were passed six times in a roll mill system at 50 °C, 5 mm gap, 32–24 rpm, and left for cooling for one hour. In a second step, the cooled composite was introduced in the mixer with 1.1 phr sulphur, 0.3 phr MBT, 3.2 phr DPG, and 2.3 phr CBS at 60 °C, 40 rpm for 2 min. Another six passes in the roll mill system were performed and the nanocomposite was finally cured under a hot press at 170 °C, 150 Bars for 10 min.

As the composition of all samples was identical, independently of the type of filler used, we assumed that the degree of crosslinking by sulphur in all nanocomposites was constant. For this reason, it is not discussed in the paper.

## 3. Results and Discussion

**Figure 1:**
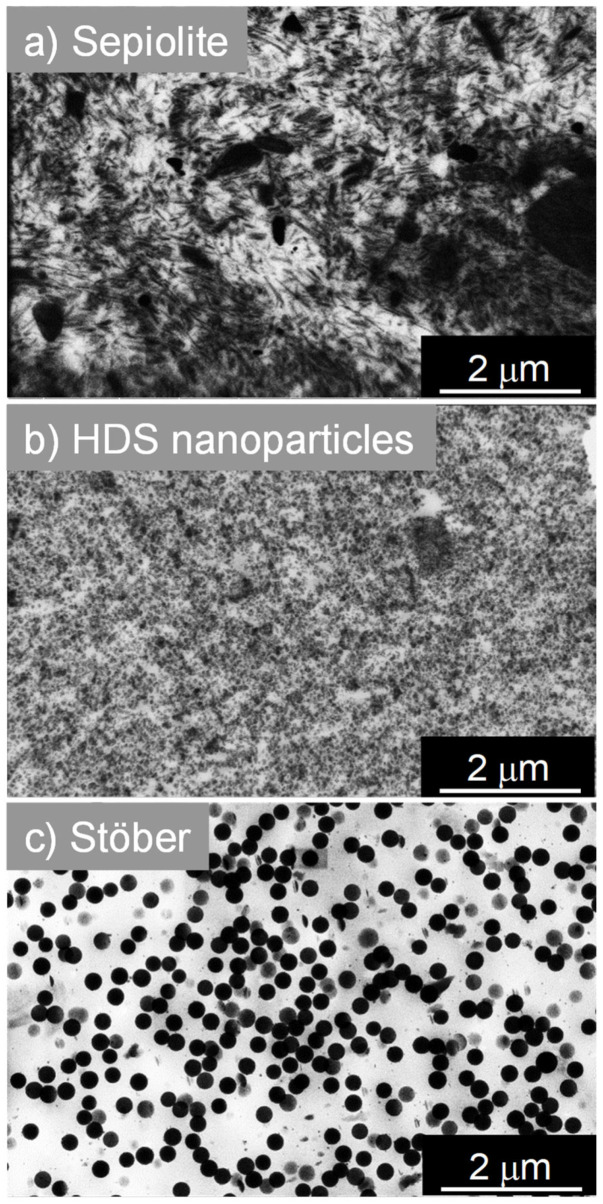
STEM images of reference nanocomposites synthesized with 80 phr of one type of filler only: (**a**) Sepiolite, (**b**) HDS, and (**c**) Stöber nanoparticles (250 nm).

The influence of spherical/spherical and spherical/anisotropic dual fillers in nanocomposites has been studied through nine samples prepared with precipitated silica HDS nanoparticles (used as the major filler in dual filler nanocomposites, forming fractal silica aggregates), silica Stöber nanoparticles of 250 nm diameter, and Sepiolite silica nanorods as fillers. The classification and filling degree of the samples studied within this work is indicated in Table 2. Three composites have been prepared with 80 phr only one type of silica filler (i.e., HDS, or Stöber nanoparticles of 250 nm diameter, or Sepiolite silica nanorods) as references (Figure 1).

**Figure 2:**
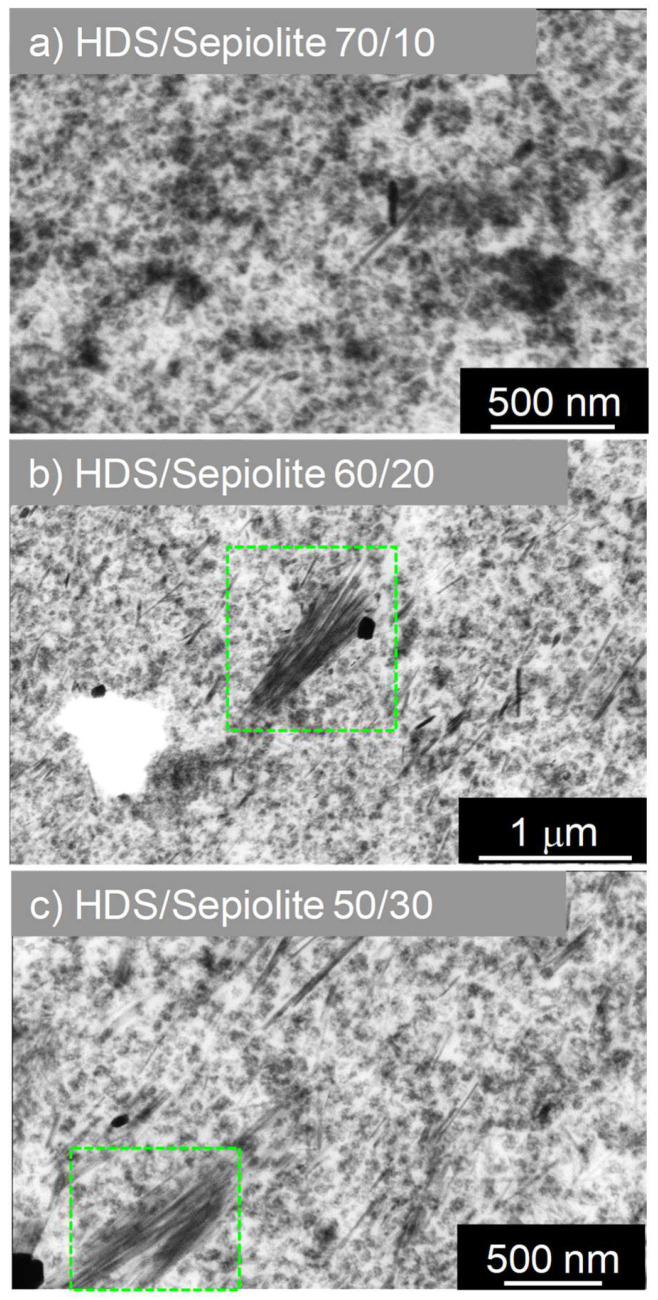
STEM images of nanocomposites synthesized with dual filler: (**a**) HDS/Sepiolite-70/10, (**b**) HDS/Sepiolite-60/20 (60/20), (**c**) HDS/Sepiolite-50/30.

Three nanocomposites have been prepared with different ratios of fractal/anisotropic fillers HDS/Sepiolite: respectively, 70/10 phr, 60/20 phr, and 50/30 phr (Figure 2).

**Figure 3:**
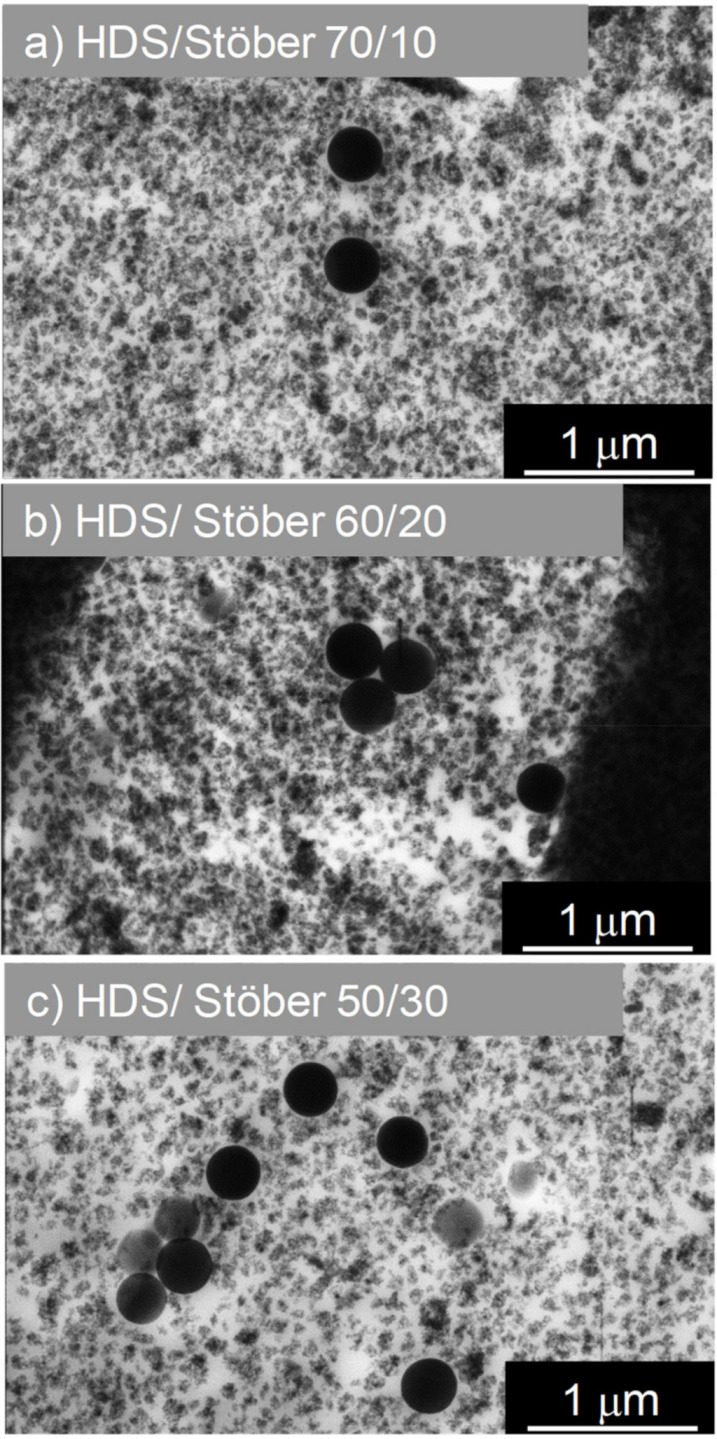
STEM images of nanocomposites synthesized with dual filler: (**a**) HDS/Stöber-70/10, (**b**) HDS/Stöber-60/20, (**c**) HDS/Stöber-50/30.

Three other samples have been prepared with different ratios of spherical/spherical fillers HDS/Stöber nanoparticles 250 nm: respectively, 70/10 phr, 60/20 phr, and 50/30 phr (Figure 3).

The total silica filler charge in all nanocomposites was kept constant at 80 phr. We can clearly distinguish on STEM pictures reported in Figure 1 the spatial distribution of silica fillers in composites. In the case of nanocomposites prepared with Sepiolite nanorods (Figure 1a), HDS nanoparticles (Figure 1b), or Stöber nanoparticles (Figure 1c) as single filler, there is no observable preferential orientation for any kind on filler. Fillers are randomly dispersed in the polymeric matrix. Concerning the percolation threshold of fillers in those nanocomposites, the two samples containing 80 phr of HDS nanoparticles and Sepiolite seem to outreach this percolation point within the rubber matrix. Together with well-distributed material, Sepiolite already shows bundles of silicate nanorods which may be due to an insufficient de-lamination or exfoliation-like process in the mixer. HDS clusters, beyond the percolation threshold, are in contact and seem to form an agglomerate exhibiting a uniform filler background. On the contrary, in the case of Stöber 80 phr, the particles are well dispersed and do not form a percolating network. Stöber nanoparticles are 250 nm diameter. They are approximately twenty times bigger than HDS nanoparticles or than the Sepiolite width. Consequently, an equal “mass” of 80 phr of Stöber nanoparticles in the rubber corresponds with a smaller number of nanoparticles per unit volume compared to HDS or Sepiolite. The same conclusion applies to the overall silica surface area that is much lower for Stöber NPs than in the case of HDS. Those two behaviours explain the non-percolating state of Stöber nanoparticles in the compound (Figure 1c). When HDS nanoparticles and anisotropic Sepiolite (Figure 2) are mixed as dual fillers in a ratio of 70/10 (Figure 2a), the structure looks very similar to the HDS single-filler nanocomposite, but few dispersed Sepiolite needles are visible within the structure. Increasing the amount of Sepiolite in the compound, i.e., HDS/Sepiolite-60/20 (Figure 2b) and HDS/Sepiolite-50/30 (Figure 2c), leads to the formation of bundles of Sepiolite in the matrix (highlighted with green boxes). Interestingly, they are showing a preferential orientation toward one direction. It is worth mentioning that the orientation of the Sepiolite rods was not observed in absence of Silica HDS (Figure 1a). The presence of HDS is apparently responsible for this Sepiolite alignment. Having oriented structures within the composite at the nanometric/micrometric scale could lead to improved macroscopic properties of the nanocomposite. Dual spherical/spherical fillers are presented in the Figure 3, with ratios of HDS/Stöber nanoparticles of 70/10 (Figure 3a), 60/20 (Figure 3b), and 50/30 (Figure 3c). Those STEM images show dispersed Stöber and HDS nanoparticles in the rubber matrix. On the picture Figure 3c, it appears that the replacement of 30 phr of HDS with larger Stöber nanoparticles prevents the percolation of HDS fillers in the rubber matrix.

### 3.1. Dynamic Mechanical Analysis

**Figure 4:**
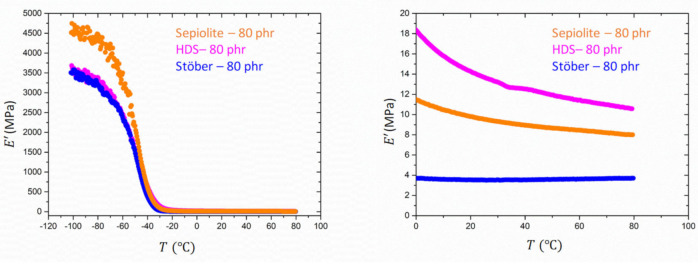
Evolution of $E^{\prime }$ with temperature for the reference SBR samples, HDS-80 phr (magenta), Sepiolite-80 phr (orange), and Stöber-80 phr (blue).

DMA analysis has been performed in order to determine mechanical properties of nanocomposites. Figure 4 shows the evolution of the storage modulus $E^{\prime }$ with the temperature in a range from −120 °C and 80 °C at the frequency of 1 Hz for the reference SBR samples containing, respectively, HDS-80 phr, Sepiolite-80 phr, and Stöber-80 phr with same filling degree.

In the rubbery state, the sample HDS-80 phr shows a higher elastic modulus compared to the other references. This result could be explained considering that fractal silica particles as HDS tend to form aggregates and agglomerates. This hierarchical structure, depending on the extension of the filler network, can occlude a large amount of rubber, leading to an immobilization of the occluded portion of the matrix and therefore to an increase of the stiffness in respect to the case of a dispersed system. The occluded and immobilized rubber is responsible for the increase of the effective volume fraction of the rubber. Despite the different geometry of the Sepiolite fillers, characterized by a more rod-like shape, sample Sepiolite-80 phr exhibits a lower value of the elastic modulus in the high temperature range. Rod-like silica fillers are expected to enhance the reinforcement with respect to the unaggregated spherical ones. The foreseen reinforcement of non-interacting spherical particles follows the Einstein prediction, which depends linearly on the volume fraction ϕ  but does not depend on the size, as obtained from f=1+2.5 ϕ where f is the reinforcement factor, i.e., the ratio of the reinforced modulus over the unfilled modulus. For rods, a similar expression is known, with $f=1+\left(2/3\right)\;p\phi$ [52]. Here, p represents the aspect ratio as explained above. The thinner the rod is or the longer it is, the more reinforcement is expected. Only for values of p~4, rods cannot be distinguished from spheres. The formation of a network due to an alignment of the fibers, as reported in the literature, could lead to an increase of the trapped rubber between layers of fibers [40]. However, in our case, the formation of clusters in the case of Silica HDS-80 phr seems to have a dominant effect on the reinforcement in comparison to the anisotropic fillers. The higher reinforcement of HDS, although rods should be more efficient given the aspect ratio, is caused by the aggregate morphology which expresses itself in a corresponding Payne effect. Higher moduli are due to the additional intra- and inter-aggregate bonds. The STEM analysis, indeed, did not reveal a preferential orientation of the Sepiolite anisotropic fillers in the SBR rubber in absence of HDS. The lowest modulus was instead observed for the third reference sample analyzed: Stöber-80 phr. In this case, the isotropic spherical fillers (Stöber particles) are known to give rise to a highly dispersed system, reducing, therefore, the immobilization of the polymer at the filler–matrix interphase [53]. This last sample exhibits the lowest value of the storage modulus in the rubbery regime.

**Figure 5:**
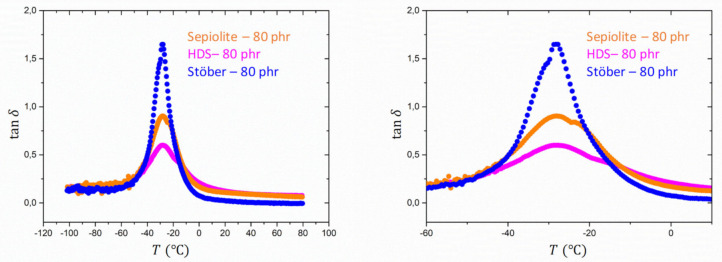
Evolution of tanδ for reference 80 phr SBR samples filled with Silica HDS-80 phr (magenta), Sepiolite-80 phr (orange) and Stöber-80 phr (blue).

The values of the elastic modulus for the different types of fillers are corroborated by the trend of tanδ as a function of the temperature. All samples, as shown in Figure 5, show a prominent peak at ~−25 °C, attributed to the glass transition. The sample Silica HDS-80 phr shows the lowest intensity peak at $T_{g}$. An intermediate peak intensity is observed for sample Sepiolite-80 phr followed by sample Stöber-80 phr, which exhibit the stronger dissipation in proximity of the transition temperature. This behaviour is explained considering the increase of the polymer chain amount involved in the transition. In the case of aggregating fillers like HDS, the degree of freedom of the polymer chains seems to be significantly decreased respective to the case of spherical particles as well as rod-like fillers.

A second peak with lower magnitude is observed for sample Silica HDS-80 phr and Sepiolite-80 phr at higher temperatures than $T_{g}$. This peak could be related to the slower relaxation process of the polymer segments bounded at the rubber-fillers interphase, characterized by a higher activation energy. The position of these peaks confirms the trend observed for the $T_{g}.$ Sample Stöber-80 phr shows instead one dominant peak corresponding to $T_{g}$, possibly due to the very low amount of bounded rubber at the matrix–filler interphase [54].

The dual filler samples are now analysed in comparison to the references. Silica HDS has been gradually substituted, respectively, by Sepiolite and Stöber particles in order to obtain dual filler systems with total loading of 80 phr.

**Figure 6:**
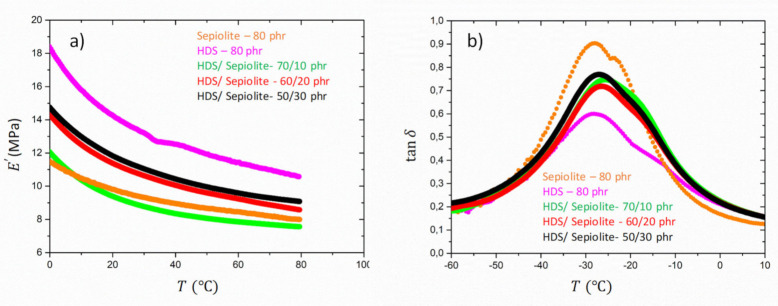
Plot of $E^{\prime }$ (**a**) and tanδ (**b**) versus temperature for reference SBR samples and dual fillers Sepiolite/HDS-10/70 phr, Sepiolite/HDS-20/60 phr, Sepiolite/HDS-30/50 phr.

Three different dual filler SBR samples were analysed with the same filling degree. Figure 6 shows the storage modulus and the loss factor tanδ as a function of the temperature for Sepiolite/HDS dual filler compounds. Despite the lower modulus at high temperature observed for the Sepiolite-filled rubber compared to the HDS one, the increase of the Sepiolite content in the dual-filler system yields an increase of the storage modulus in the temperature range between 0 and 80 °C This evidence could be attributed to the cooperative effect of the two types of fillers. If 10 phr of HDS is replaced by Sepiolite, a strong drop of the modulus is observed in the rubbery regime. However, this drop seems to be compensated by the increase of the Sepiolite amount in the other dual filler compounds. The addition of a higher amount of Sepiolite in the dual filler compounds leads, as observed, to an increase of $E^{\prime }$ even though the sample containing HDS only exhibits the highest modulus value in the rubbery regime. This increase could be explained by considering the preferential arrangement of the anisotropic filler in the presence of the fractal one. The arrangement seems to be dependent on the amount of rod-like fillers in the dual filler mixture and is observed to be more effective for the compounds containing, respectively, 20 and 30 phr of Sepiolite (see Figure 2). The substitution of HDS with 10 phr of Sepiolite could, at first, give rise to a decrease of the stiffness of the compound, as the rod-like filler might induce a breakage of the HDS clusters. The increment of Sepiolite amount at the expenses of silica content in the case of HDS/Sepiolite-60/20 and HDS/Sepiolite 50/30 could, however, restore a more uniform mixture where now percolation of both silica and Sepiolite fillers occur and, because of an induced preferential orientation of the rods, yield a higher elastic modulus. In addition, during the mixing steps (internal mixer and roll mill), the formation of such a percolating network can play a key role in the Sepiolite orientation.

The mixing energy required at the mixing/milling steps is higher when percolation is reached (see Table S1 in supporting information). As a consequence, the shearing forces applied to the compound will increase and those forces can be highly oriented, especially during the mixing or roll mill steps.

The evolution of tanδ (Figure 6) with the temperature is also analysed. In this case, the two peaks corresponding, respectively, to $T_{g}$ and to the dynamic of the bounded rubber at the interphase are not well separated. The increased Sepiolite amount is not reflected by a shift of the secondary peak intensity as expected from the increase of the storage modulus. This result can be explained by the different structural arrangement occurring in the mixture of two different types of fillers with different geometry. The orientation of Sepiolite in the presence of percolating fillers such as HDS, as evidenced in Figure 2b,c, could, on the other hand, affect the specific arrangement of the Silica HDS clusters as a consequence of a cooperative effect. The preferential orientation of silica filler clusters, which is known to significantly affect the hysteresis behaviour as well as the elastic modulus of the rubbery compounds, is, however, not highlighted by the STEM images in our case. [55].

**Figure 7:**
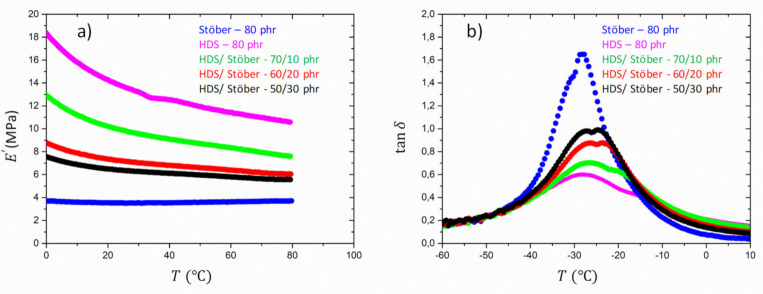
Plot of $E^{\prime }$ (**a**) and tanδ (**b**) versus temperature for reference SBR samples and dual fillers Scheme 10. phr (R-31), Stöber/HDS-20/60 phr (R-32), Stöber/HDS-30/50 phr (R-33).

A different trend is instead reported for the mixtures of HDS and Stöber particles. Figure 7 shows the storage modulus and the loss factor tanδ as a function of the temperature for Stöber/HDS dual filler compounds.

The evolution of $E^{\prime }\;$for the reference samples and the mixtures shows that the increase of the Stöber content in the dual-filler systems yields a decrease of the elastic modulus.

This behaviour could be attributed to the lower tendency of Stöber particles to form aggregates, as shown in the STEM pictures in Figure 3. A cooperative effect of the two fillers is, however, envisaged in this system, too. The higher content of Stöber particles in the mixture leads to a higher dispersion of the fillers in the rubber and, therefore, to a lower constraint exerted by the fillers on the rubber. The lower fraction of aggregates formed by HDS fillers is reflected in a smaller amount of bounded rubber. The reduction of aggregates formation with the substitution of HDS with Stöber particles has been confirmed by the STEM analysis. Figure 3 reveals that the decrease of HDS in the dual fillers system causes a different dispersion of the clusters and that the Stöber particles do not mix themselves into the aggregates due to their size, but position themselves in between agglomerates, therefore reducing the network. This leads to a lower modulus and can be compared to the effects of adding a non-interacting solvent to rubbers. This effect is not compensated by the increased amount of the spherical Stöber particles that are not forming aggregates and do not contribute to the reinforcement.

This observation is confirmed by the analysis of tanδ as a function of the temperature (Figure 7). The dual-filler compounds show a clear distinction between the main peak corresponding to the glass transition and the secondary peak at higher temperature related to the relaxation of the bounded rubber. A shift of the secondary peak to lower temperatures as well as an increase of its magnitude is observed with the increase of Stöber content in the mixture, indicating a lower constraint at the filler-rubber interphase.

For the dual filler systems, a cooperative interaction between the two types of fillers is observed. This synergic interaction, leading to a change in the elastic modulus in the rubbery regime, is found to be dependent on the geometry of the fillers as well as on their ability of forming hierarchical structures.

### 3.2. Tensile Tests

**Figure 8:**
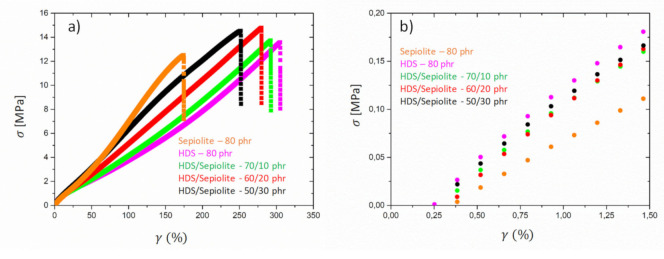
Stress-strain curves for mixtures of Sepiolite and HDS Silica dual filler compounds in the full deformation range (**a**) and at low deformation (**b**).

Figure 8 shows the stress-strain curves obtained for the mixtures of HDS and Sepiolite and the two reference samples containing only one type of filler. First, the analysis of the stress at low deformation is indicating that the sample SBR HDS 80 phr has a higher modulus than SBR Sepiolite-80 phr. A value of $E^{\prime }\sim 14\;\mathrm{MPa}$ was estimated for the sample HDS Silica-80 phr, while for the other reference, Sepiolite-80 phr, a value of $E^{\prime }\sim 10\;\mathrm{MPa}$ was found. These values seem to be approximately in agreement with the DMA values observed in Figure 6 at room temperature. For the mixtures, however, a value of $E^{\prime }\sim 13\;\mathrm{MPa}$ was observed, although the DMA experiments revealed clear differences among the moduli in the rubbery regime and, specifically, a significant drop when 10 phr of HDS silica were replaced by Sepiolite. To explain this discrepancy, the different sensitivity of the two techniques has to be taken into account. The tensile test does not allow a clear distinction of the moduli for the mixtures at very low strain as in the case of DMA test, which is instead carried out with a small deformation.

The high strain behaviour reveals the highest stress value for the SBR Sepiolite-80 phr, while a decrease is observed for the dual filler blends when the amount of Sepiolite decreases. The sample SBR HDS-80 phr shows the lower stress value at higher deformation. On the other hand, the value of elongation at break follows the opposite trend. These different behaviours could be explained by considering that, despite the higher reinforcement at low strain conferred by the silica HDS filler, the gradual strain induced by the orientation of the rods gives rise to a strain hardening of the rubbers containing the Sepiolite filler. This observation would also explain the decrease of the elongation at break with the increase of the Sepiolite amount. The rigid rods orienting along the deformation direction at high strain might cause a reduction of the rubber elongation ability.

**Figure 9:**
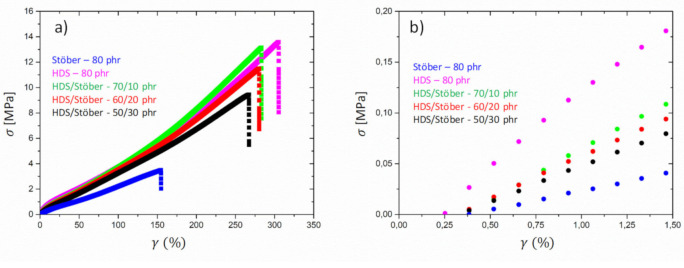
Stress-strain curves for mixtures of Stöber and HDS Silica dual filler compounds in the full deformation range (**a**) and at low deformation (**b**).

Different behaviour was observed for the dual filler compounds containing Stöber particles and Silica HDS. The spherical Stöber filler was found to “dilute” the aggregates formed by HDS particles, leading to a decrease of the elastic modulus in the rubbery regime, as highlighted by SEM pictures in Figure 3. Stress-strain curves for dual fillers samples containing Stöber and Silica HDS particles are reported in Figure 9. The low strain regime shows the highest reinforcement for the sample SBR HDS-80 phr, while the other reference sample SBR Stöber-80 phr exhibits the lowest stress in the whole deformation range and the lowest elongation at break, with an estimated modulus of $E^{\prime }\sim 3\;\mathrm{MPa}$. The dual filler compounds show that the increase of the Stöber amount in the mixtures leads to a decrease of the stress value. For the three mixtures containing increasing amounts of Stöber from 10 to 30 phr, the elastic modulus was estimated as $E^{\prime }\sim 10\;\mathrm{MPa}$, $E^{\prime }\sim 8\;\mathrm{MPa}$, and $E^{\prime }\sim 7\;\mathrm{MPa}$, respectively. A decrease of the elongation at break was also reported with the increase of the Stöber content in the case of Stöber/HDS-20/60 and Stöber/HDS-30/50. Although the Stöber particles were found to be responsible for a decrease of the modulus at the rubbery regime in DMA and for the reduction of the fraction of immobilized rubber, the increase of these particle amounts induces an increase of the rubber brittleness. The reason could be related to the fact size of the Stöber particles, which, as shown by the STEM figures (see Figure 1), are significantly bigger than the average size of HDS clusters. At high deformation, while the clusters are subjected to the strain due to a certain amount of bounded rubber, the Stöber particles are acting as undeformed cross-links. This observation could explain the lower elongation at break with the increase of Stöber amount, despite their higher dispersibility.

## 4. Conclusions

This work has been dedicated to the study of mechanical properties of SBR-based nanocomposites containing spherical or anisotropic dual fillers. Fractal/spherical and fractal/anisotropic dual filler nanocomposites were prepared with HDS nanoparticles/Stöber nanoparticles and HDS nanoparticles/Sepiolite nanoneedles, respectively. We highlight, using STEM and DMA analysis, that, in the system Sepiolite/HDS, both fillers work cooperatively, and the HDS induces a preferential alignment of Sepiolite clusters in the rubber matrix. We hypothesize that the orientation of the Sepiolite is activated by the presence of a HDS percolating network, and the resulting oriented forces which will occur at the mixing steps. The induced preferential orientation gives rise to an increase of nanocomposites’ storage modulus in the rubbery regime, as by the DMA. The tensile tests carried out at a higher deformation range on the same samples evidenced a reduction of rubber elongation ability with the increased amount of nanorods in the mixture, indicating an orientation of the rods along the deformation direction induced this time by the high strain applied. On the other hand, in the Stöber/HDS dual filler system, the spherical component seemed to show more of a diluting effect than a reinforcing one. This observation is associated with the trend of tan δ as a function of temperature in DMA, which clearly shows a decrease of the immobilized rubber with the increase of Stöber amount in the mixture. At the same time, a decrease of the elastic modulus at high temperature with the increase of Stöber concentration has been reported. An apparent contradiction with the lower elongation at break observed for the mixtures containing a higher amount of Stöber particles could instead be explained by considering the non-deformability of these spherical fillers that might act as permanent cross-links under the condition of high strain, leading to an earlier rupture of the rubbery material.

Based on those observations, the dual filler system fractal/anisotropic silica-based structure appears as a promising solution to reach reinforced nanocomposites. As raw Sepiolite fibres or nanoneedles can differ in quality, composition, structure, and surface state depending on their origin, it may be worth studying the behaviour of dual filler nanocomposites prepared with anisotropic silica nanorods instead of Sepiolite.

## Figures and Tables

**Table 1:** Nano-composite composition in phr.

Element	Element Concentration (phr)
SBR	80
PBD	20
TDAE Oil	25
Zinc Oxide	2.5
Stearic Acid	3
6PPD	2.5
TESPD	8
Sulfur	1.1
MBT	0.3
DPG	3.2
CBS	2.3
**Silica-based filler**	**80**

**Table 2:** Sample classification-filler amount in SBR rubber matrix.

Sample	HDS (phr)	Sepiolite (phr)	Stöber (phr)
HDS-80 phr	80		
Sepiolite-80 phr		80	
Stöber-80 phr			80
HDS/Sepiolite-70/10	70	10	
HDS/Sepiolite-60/20	60	20	
HDS/Sepiolite-50/30	50	30	
HDS/Stöber-70/10	70		10
HDS/Stöber-60/20	60		20
HDS/Stöber-50/30	50		30

## Supplementary Materials

The following are available online at https://www.mdpi.com/article/10.3390/polym13152413/s1, Table S1: Mixing energies required to process nanocomposites with different fillers.
