# Supplementary material for: Hybrid Silica-Based Fillers in Nanocomposites: Influence of Isotropic/Isotropic and Isotropic/Anisotropic Fillers on Mechanical Properties of Styrene-Butadiene (SBR)-Based Rubber
Source: Polymers (Basel). 2021 Jul 22;13(15):2413. doi: 10.3390/polym13152413 (PMC8348337; doi:10.3390/polym13152413)
Supplement: Supplementary file 1 [file polymers-13-02413-s001.zip › polymers-1284111-supplementary.pdf]

## Supporting information

Table S1: Mixing energies required to process nanocomposites with different fillers.

| Sample | Energy (kJ) |
|---|---|
| 200 MP – 80 phr | 80 |
| Sepiolite – 80 phr | 55 |
| Stöber – 80 phr | 50 |
| 200 MP/ Sepiolite – 70/10 | 75 |
| 200 MP/ Sepiolite– 60/20 | 73 |
| 200 MP/ Sepiolite– 50/30 | 68 |
| 200 MP/ Stöber– 70/10 | 68 |
| 200 MP/ Stöber – 60/20 | 63 |
| 200 MP/ Stöber – 50/30 | 58 |
